# Effect of suckler cow breed type and parity on the development of the cow-calf bond post-partum and calf passive immunity

**DOI:** 10.1186/s13620-024-00276-x

**Published:** 2024-07-05

**Authors:** Noeleen Brereton, Mark McGee, Marijke Beltman, Colin J. Byrne, David Meredith, Bernadette Earley

**Affiliations:** 1 Teagasc, Grange, Animal & Grassland Research and Innovation Centre, Dunsany, Co. Meath Ireland; 2 Teagasc Rural Economy Development Programme, Dublin 15, Ashtown, Co. Dublin, Ireland; 3 School of Veterinary Medicine, University College Dublin, Belfield, Dublin 4, Co. Dublin, Ireland

**Keywords:** Beef cow, Colostrum, Maternal behaviour, Natural suckling, Oro-esophageal feeder, Artificially-fed colostrum, Genotype, Passive immunity measures

## Abstract

**Background:**

Development of the cow-calf bond post-partum and passive immunity of calves from spring-calving beef × beef (B×B) and beef × dairy (B×D) cow genotypes was determined using primiparous and multiparous (Experiment 1), and primiparous and second-parity (Experiment 2) animals. In Experiment 1, calves either suckled colostrum naturally (‘natural-suckling’) (*n* = 126), or were fed colostrum, using an oesophageal-tube (‘artificially-fed’) (*n* = 26), from their dam within 1-h post-partum. In Experiment 2, all calves (*n* = 60) were artificially-fed colostrum from their dam. Prior to colostrum suckling/feeding, colostrum was sampled for IgG analysis. The cow-calf bond was assessed using CCTV recordings during the first 4-h post-partum. Calves were blood sampled at 48-h post-partum to determine IgG and total protein (TP) concentrations, and zinc sulphate turbidity (ZST) units.

**Results:**

There was no difference (*P* > 0.05) in cow licking behaviours and calf standing and suckling behaviours between the genotypes, except in Experiment 2 where B×D calves had more attempts to suckle before suckling occurred (*P* ≤ 0.05) compared to B×B calves. In Experiment 1, multiparous cows licked their calves sooner (*P* ≤ 0.05) and for longer (*P* < 0.01), and their calves had fewer attempts to stand (*P* < 0.001), stood for longer (*P* = 0.05), and had fewer attempts to suckle before suckling occurred (*P* < 0.001) than primiparous cows; there was no parity effect on cow-calf behaviour in Experiment 2. Colostrum IgG concentrations and measures of calf passive immunity did not differ (*P* > 0.05) between the genotypes in either Experiment. In Experiment 1, colostrum IgG concentrations were greater (*P* ≤ 0.05) in multiparous compared to primiparous cows and their calves had superior (*P* ≤ 0.05) passive immunity; no effect of parity was found in Experiment 2. Passive immunity did not differ (*P* > 0.05) between suckled and artificially-fed calves in Experiment 1.

**Conclusions:**

Cow genotype had little effect on cow-calf behaviours, but under ‘natural-suckling’ conditions primiparous cows expressed maternal inexperience and their calves were less vigorous than multiparous cows. Colostrum IgG concentration and calf passive immunity measures were unaffected by genotype, but under ‘natural-suckling’ conditions calves from primiparous cows had lower passive immunity.

**Supplementary Information:**

The online version contains supplementary material available at 10.1186/s13620-024-00276-x.

## Introduction

Irish suckler beef production is primarily based on seasonal spring-calving, grass-based systems using predominantly late-maturing (‘continental’) cow and sire breeds [1]. Cow replacement strategy comprises of heifers sourced from within the suckler beef herd (beef × beef, B×B) and, to a lesser extent, sourced from within the dairy herd (beef × dairy, B×D) [1, 2]. Within these production systems, cows are usually accommodated indoors during the ‘winter’ period with calving also occurring indoors (February to April) corresponding with the start of grass growth in spring [3].

The cow-calf bond is formed following parturition, and maternal behaviour post-calving influences calf survival, health and pre-weaning performance [4–6]. To initiate and form the bond, the dam licks and sniffs the calf coupled with low-pitched vocalisation [7, 8]. Additionally, the licking motion stimulates the calf to stand and suckle the dam [4]. Learning to suckle quickly is vital as the neonatal calf is born with an underdeveloped immune defence, and obtains passive immunity through ingestion of colostrum, which provides immunologic protection during the first two to four weeks of life until it is able to generate its own adaptive immune response [9]. Beef calves with low passive immunity are at greater risk of morbidity and mortality, and have poorer growth performance [10–12], which has direct and indirect economic costs [13].

The intestinal sites that absorb immunoglobulins (Ig) or antibodies begin to close when the calf is born and by six hours of birth the capacity for the calf to absorb immunoglobulin G (IgG) through the intestinal wall has decreased by 50% with complete gut closure to absorption of IgG by approximately 24-hours post-partum [14]. Consequently, factors that impinge upon the duration between birth and first-suckling negatively impact on calf passive immunity and, in this regard, the rapid development of a good cow-calf bond is essential [14]. To ensure a newborn calf receives colostrum, an alternative to suckling is artificial-feeding of the dam’s colostrum using either an oesophageal tube or teat-bottle [15, 16]. This colostrum management practice is advantageous as a known volume can be fed in a timely manner [17]. Despite the importance of prompt colostrum consumption by the newborn suckler calf [15], there is comparatively little published literature pertaining to the suckling-related behaviour of beef calves from multiparous and primiparous dams post-partum, and how this behaviour affects calf passive immunity. This information deficit is particularly apparent vis-à-vis the cow genotypes and ‘indoor’ calving conditions prevalent in Ireland, as well as under contrasting colostrum management practices.

In a recent Irish large-scale on-farm study Todd et al. [10] found that failed transfer of passive immunity (FTPI) of immunity, based on ELISA ‘cut-offs’ (serum IgG < 9 mg/ml), was prevalent in 31% of suckler beef (encompassing both B × D and B × B) calves. Similarly, in a study of 84 farms in Great Britain, 15% of suckler calves were classified as having FTPI (IgG, < 10 mg/ml using radial immunodiffusion, (RID)) and 37% were classified as having inadequate (IgG, < 24 mg/ml) passive immunity [18]. Likewise, research using primiparous Charolais cows on two experimental farms in France, Martin et al. [11], found that 22% and 10% of calves had IgG_1_ concentrations < 10 mg/ml using RID and ELISA tests, respectively. These findings are also broadly consistent with recent Canadian studies which indicated that on commercial beef farms between 18% and 35% of calves had inadequate (RID - IgG, < 24 mg/ml) passive immunity [19, 20].

In the review paper by McGee and Earley [9]), differences between cow genotypes and parity in colostrum immunoglobulin concentration and calf passive immunity are reported; however, the effects across published studies are inconsistent. For example, McGee et al. [1] found that colostrum IgG concentrations were greater in BxD cows compared to Charolais cows, and calves born to B×D cows had greater serum Ig concentrations and ZST units at 48 h post-partum than calves born to Charolais cows. In contrast, Murphy et al. [21] found no difference in colostrum IgG concentrations between Charolais and Limousin or B×D cow breed types but, likewise, calves from B×D cows had greater serum IgG concentrations and ZST units than those from Charolais and Limousin cows. Calf passive immunity levels were high in those two research centre studies [1, 21].

In terms of suckler cow parity, whereas most studies show no effect of parity on colostrum IgG concentrations [15, 22, 23], values were reported to be lower for primiparous compared to multiparous B x D cows [15, 24], similar for primiparous compared to multiparous Charolais cows [24], or lowest for primiparous and highest for third-parity suckler beef cows, with parities two, four, five and six being intermediate [25]. In commercial practice, many confounding factors can impact calf passive immunity. Consequently, there is an urgent need to re-assess the passive immunity of calves from ‘modern’ suckler cow genotypes and parity, in Ireland under standard, but controlled, conditions.

Therefore, the primary objectives of this study were to determine the effect of suckler cow genotype (B×B *v.* B×D) and parity on the development of the cow-calf bond and its relationship with calf passive immunity under conditions where calves suckled colostrum ‘naturally’ (Experiment 1), or were artificially-fed colostrum (Experiment 2), post-partum. A secondary goal was to determine the relationship between three different passive immunity tests, namely sRID, ZST and TP.

## Materials and methods

### Animals and management

Data were obtained over two years - Experiments 1 and 2 - from a spring-calving suckler cow research herd comprised of two genotypes - beef × beef (B×B) and beef × dairy (B×D) cows. All multiparous cows were artificially inseminated using Charolais or Simmental sires, and first-parity cows (bred to calve at two years of age) were artificially inseminated using easy-calving Aberdeen Angus or Hereford sires.

### Pre-partum management

Cows were accommodated within pens (7 cows/pen) in a concrete slatted floor shed at approximately 2–5 months pre-calving at a space allowance of 3.3 m^2^/animal. In both experiments, cows were offered second-harvest grass silage (*in-vitro* dry matter (DM) digestibility and crude protein concentration of 689 g/kg and 118 g/kg DM, and 679 g/kg and 146 g/kg DM in experiments 1 and 2, respectively) *ad libitum* pre-partum. In addition, all cows received vitamin and mineral supplementation (magnesium 5.9%; potassium iodide 0.001%; trace elements (Zn, 62.5 g/kg; Cu, 41.7 g/kg; Se, 0.8 g/kg; Co, 0.7 g/kg; Na, 0.5 g/kg and Mg, 0.1 g/kg) supplied through the drinking water (TerraNutritech, Ireland). A combined bovine rotavirus and E. coli vaccine (Bovilis, MSD Ireland) along with a live vaccine of Infectious Bovine Rhinotracheitis (IBR) was administered to all cows by intramuscular injection 4-to-12 weeks prior to expected calving date as part of routine husbandry management. Between 1 and 4 days prior to their expected calving date, cows were transferred to single straw-bedded pens (4.5 m × 4.8 m).

### Post-partum management

Calf births were supervised by the stockperson via direct visual observation and the use of CCTV cameras in each calving pen. Immediately post-partum all cows were momentarily restrained in a head gate to allow the stockperson to apply disinfectant to the calf navel using iodine spray (Povidone Iodine 10%, Foran Animal Health, Kilkenny, Ireland), weigh the calf, determine the ‘suckle reflex’ (Experiment 1), and to milk colostrum or obtain a colostrum sample from the cow (see below). Cows requiring assistance at birth were already restrained in the head-gate during the delivery. Once the aforementioned procedures were implemented, the cow was immediately released from the head gate and left to bond with her calf. The cow and her calf remained together in the individual straw-bedded calving pens for 24-h post-partum, and had freedom to express natural behaviour, such as the cow allogrooming her calf.

Post-partum, all cows continued to receive the same grass silage *ad libitum* as offered pre-partum, and first-parity animals were additionally supplemented with 1.5 kg of a barley-based concentrate per head daily.

### Experiment 1

In Experiment 1, a total of 78 B×B cows (Aberdeen Angus, Charolais, Limousin and Simmental crossbreds) and 74 B×D cows (Aberdeen Angus × and Limousin × Holstein-Friesian) were included. They comprised of 38 primiparous (19 B×B and 19 B×D) and 114 multiparous - parity 2 to 4 - (59 B×B and 55 B×D) cows. The calves either suckled the dam naturally (suckled, *n* = 126) with no intervention, or were fed colostrum from their dam (hand-milked without oxytocin administration) using an oesophageal-tube (Kerbl Oral Drench Bag, Albert Kerbl GmbH, Buchbach, Germany) (artificially-fed, *n* = 26). Intervention was primarily based on the strength of the calf suckle reflex, which was determined immediately post-partum by placing two fingers longitudinally into the calf’s mouth and gently rubbing the roof of the mouth [26]. Calves exhibiting a strong jaw tone with a rhythmic suckle reflex were characterised as ‘strong’, and calves with weak jaw tone or non-rhythmic suckle reflex as ‘weak’ [26]. Calves exhibiting a ‘strong’ suckle reflex were left to naturally suckle for up to 4 h. Calves were artificially-fed colostrum when they exhibited a ‘weak’ suckle reflex (*n* = 23), or if they failed to suckle naturally by approximately 4 h post-partum (*n* = 3). The mean volume of colostrum artificially-fed to calves from primiparous cows was 1.2 L (SD 0.48), equivalent to 2.8% of birth weight, and to calves from multiparous cows was 1.4 L (SD 0.43), equivalent to 3.2% of birth weight. The colostrum volume fed was constrained by the yield of the dam, and up to a maximum of 2 L was fed equivalent to less than 3.1% (SD 1.06) birth weight. Artificially-fed calves were observed by farm staff to ensure that suckling occurred in due course. Calving commenced on 2 February and ended 26 April; mean calving date was 15 March.

### Experiment 2

In Experiment 2, a total of 29 B×B cows (Aberdeen Angus, Limousin and Simmental crossbreds) and 31 B×D cows (Aberdeen Angus × and Limousin × Holstein-Friesian) were included. They comprised of 39 primiparous (17 B×B and 22 B×D) and 21 s-parity (12 B×B and 9 B×D) cows. Immediately post-partum, the cow was momentarily restrained for hand-milking colostrum to feed the calf using an oesophageal-tube as described above. The mean volume of colostrum fed to calves from primiparous and second-parity cows was 1.1 L (SD 0.54), and 1.3 L (SD 0.52), equivalent to 2.8% and 3.1% mean percentage of birth weight, respectively.

Calving commenced on 2 February and ended on 22 May; mean calving date was 27 March.

### Measurements in experiments 1 and 2

Post-partum, calving difficulty score (scale 1–5, 1 = no assistance; 2 = minor handling (‘checking’); 3 = minor assistance; 4 = mechanical assistance (calving jack) and 5 = caesarean section) adapted from McCabe et al. [2], cow docility score (scale 1–5, 1 = quiet and 5 = very aggressive) [27] and calf vigour score (the ability to stand and suckle the cow, scale 1–5, (1 = poor, 2 = very lazy, 3 = lazy, 4 = vigorous, and 5 = excellent/very vigorous), adapted from ICBF [28] were recorded, cows were weighed and body condition score (BCS) (scale 0–5, 0 = emaciated and 5 = very fat; [29] was determined within 24-hours post-partum. The cows were nutritionally managed to calve down at a moderate BCS (2.6 to 3.0) in order to reduce the incidence of calving difficulty. The newborn calves were weighed immediately after birth, as described earlier.

### Video surveillance and behaviour observation

Maternal behaviours were recorded using high definition EXIR Dome 3.66 mm infra-red cameras (Hikvision, No.555 Qianmo Road, Binjiang District, Hangzhou, China) that were connected to a network video recorder. The CCTV recordings were analysed from continuous real-time recordings, which commenced following the aforementioned post-partum cow and calf management, and colostrum sampling interventions. The ten behavioural variables observed were (i) time taken by the cow to first-licking of the calf for 15 s or longer, (ii) duration of the first-licking, (iii) total number of attempts the calf made to stand, (iv) total duration of the attempts to stand, (v) time taken by the calf to stand successfully (i.e. steady on all four legs), (vi) duration of the first-standing on all four legs, (vii) total number of attempts to suckle (i.e. calf teat-seeking in close proximity to the cows udder) before suckling occurred, (viii) duration of the attempts to suckle before suckling occurred, (ix) time taken by the calf to first-suckling, and (x) total duration of the first-suckling bout. The definitions for each behaviour were based on those described previously [7, 8, 30]. In Experiment 1, only the calves that ‘naturally’ suckled their dams within the first approximately 4-h post-partum were included in the behaviour observations (*n* = 126). In Experiment 2, the behaviour of all cow-calf pairs was observed up to the first suckling, following which observations ceased.

### Colostrum and blood sampling

A 40 ml sample of colostrum was hand-milked (the first ‘draw’ was discarded) from the most accessible front quarter of the udder directly after calving and was stored at -20 °C pending analysis. Previous research has shown that colostrum IgG concentration is similar between the quarters of the udder, and similar for within-quarter fractions [15]. Calves were blood sampled by jugular venepuncture, using an 8.5 ml SST gel clot vacutainer (Vacutainer ^®^ BD Cruinn Diagnostics, Dublin), at 48-h post-partum. Blood samples were left at room temperature for 1-h followed by a period of approximately 24-h at 4 °C to permit clotting. The samples were subsequently centrifuged (1600 × g for 10 min) and the serum samples were stored at approximately − 20 °C prior to analysis.

### Laboratory analysis

Colostrum samples were defrosted at room temperature, centrifuged (4500 × g for 20 min at 6 °C) to obtain fat-free samples. Colostrum IgG concentrations were determined using single radial immunodiffusion (sRID) test kits (Triple J Farms, Bellingham, WA, USA). Each kit was supplied with three standard controls (low, 280 mg/dl; mid, 1400 mg/dl; and high, 2800 mg/dl)) and 5 µl of each was applied to the plate. Colostrum samples were diluted appropriately (1:3 to 1:8) using 0.9% NaCl to fall within the range of the standard curve. Plates were incubated for 24-h at room temperature to obtain an end-point reading. The diameters of the precipitin rings were measured using a digital callipers (RS PRO Digital Metric Vernier Calliper, Radionics LTD, Dublin). The diameters of the precipitin rings were plotted against the IgG concentrations of the standards to calculate the slope of the line, and the concentrations of the samples (colostrum and serum) were computed from this.

Calf serum samples were analysed using ‘direct’ and ‘indirect’ methods to determine passive immunity. The direct test used to determine calf serum IgG concentrations was sRID, as described earlier, with the exception that the serum dilution rates were 1:2. The two indirect passive immune tests used on calf serum samples were the zinc sulphate turbidity (ZST) and total protein (TP). The ZST method was carried out on serum samples at 20 °C as described by McEwan et al. [31]. Briefly, a control (100 µL of serum and 6 mL of distilled water) and test samples (100 µL of serum and 6 mL of zinc sulphate (0.208 g/L) were prepared and incubated at room temperature for 1 h; samples were transferred into cuvettes and read using a spectrophotometer at 520 nm. The TP concentrations were determined in calf serum samples using a digital hand-held refractometer with automatic temperature compensation (DR-303, Index Instruments Ltd, Cambridgeshire, UK). Before testing each sample, the refractometer was calibrated to zero using distilled water [32].

### Statistical analysis

Statistical analysis of data was applied to investigate the effects of cow genotype and parity on measures of immediate postnatal cow-calf behaviour and calf passive immunity. All statistical analysis were performed using SAS software Version 9.4 (SAS Institute Inc. Cary, NC, USA) [33]. Behavioural data (including time to first-licking the calf (sec), total duration of first-licking (sec), total number of attempts to stand by the calf, total duration of the attempts to stand (sec), time to calf standing on all fours (min), duration of the first standing on all fours (sec), total number of attempts to suckle before suckling occurred, total duration of the attempts to suckle before suckling occurred (min), time to first-suckle (min), duration of the first-suckling bout (sec)), cow weight, calf birthweight, colostrum IgG, calf serum IgG, ZST, TP were tested for normality and homogeneity of variance by histograms, q-q plots, and formal statistical tests (Shapiro-Wilk, Kolmogorov-Smirnov, Cramer-von Mises, and Anderson-Darling) as part of the UNIVARIATE procedure of SAS 9.4. Data were analysed using the PROC MIXED (mixed-effects linear models) procedure of SAS 9.4 (SAS Institute Inc., Cary, NC) with animal as the experimental unit. The statistical multivariable model had terms for cow genotype, parity, calf vigour, calf sex and their interactions with pen as a random term. Calf sex interaction term was not statistically significant (*P* > 0.10) and was subsequently excluded from the final model. The type of variance-covariance structure used was chosen depending on the magnitude of the Akaike information criterion (AIC), and the lowest AIC co-efficient was then selected. Differences between genotypes, parity and interactions were determined by F-tests using Type III sums of squares. Least-squares means are reported with standard errors. The PROC CORR procedure in SAS 9.4 was used to determine Spearman correlation coefficients between 1), calf passive immunity measures, and 2), maternal bond behavioural variables with calf passive immunity measures. Values were considered statistically significant at *P* < 0.05.

## Results

### Experiment 1

Cow live weight, BCS, docility score and calving difficulty score, and calf birth weight and calf vigour score of animals enrolled in Experiment 1 are shown in Table 1.

**Table 1:** Means (SD) for beef × beef (B×B) and beef × dairy (B×D) cow live weight, body condition score (BCS), docility score and calving difficulty score, and calf birth weight and calf vigour for the suckled and artificially-fed groups collected post-partum in experiment 1

	Suckled (*n* = 126)		Artificially-fed (*n* = 26)	
	B×B	B×D	B×B	B×D
Cow weight (kg)	637 (74.5)	604 (73.5)	609 (61.9)	548 (68.6)
Cow BCS (0–5)^1^	3.0 (0.24)	2.7 (0.24)	2.9 (0.22)	2.7 (0.30)
Cow docility score (1–5)^2^	1.3 (0.84)	1.1 (0.28)	1.1 (0.34)	1.0 (0.00)
Calving difficulty score (1–5)^3^	1.3 (0.61)	1.4 (0.71)	2.8 (1.39)	2.3 (1.06)
Calf birthweight (kg)	45.5 (5.82)	45.3 (5.84)	44.7 (7.35)	40.9 (6.70)
Calf vigour (1–5)^4^	4.9 (0.49)	5 (0.18)	3.9 (1.06)	3.9 (1.10)

^1^Cow BCS scale; 0–5 (0 = emaciated and 5 = very fat)

^2^Cow docility scale; 1–5; (1 = excellent and 5 = very aggressive)

^3^Calving difficulty score scale 1–5; (1 = no assistance; 2 = minor handling (‘checking’); 3 = minor assistance; 4 = mechanical assistance (calving jack) and 5 = caesarean section, the highest score was assigned to deliveries requiring veterinary assistance)

^**4**^Calf vigour scale; (1 = poor, 2 = very lazy, 3 = lazy, 4 = vigorous, and 5 = excellent/very vigorous)

### The effect of cow genotype and parity effects on maternal bond formation and calf passive immunity (suckled)

The effect of genotype and parity on maternal behaviours and IgG concentration are presented in Table 2. There was a genotype × parity interaction (*P* < 0.01) for time to standing whereby B×D calves from multiparous cows stood earlier (47 min) than calves from primiparous cows (81 min) but no effect of parity (60 *v.* 52 min., respectively) for B×B calves. No effect of genotype (*P* > 0.05) was found for the remaining cow-calf maternal behaviours, or on colostrum or calf passive immunity measures. Multiparous cows licked their calves sooner (*P* = 0.05), and for longer (*P* < 0.01) than primiparous cows. Calves from multiparous cows made fewer attempts to stand (*P* < 0.001), they stood for longer (*P* < 0.05) and had fewer attempts to suckle (*P* < 0.001) before suckling occurred than calves from primiparous cows. Multiparous cows had greater (*P* < 0.05) colostrum IgG concentrations and their calves had greater (*P* = 0.05) serum IgG concentrations, ZST units and TP concentrations than primiparous cows.

**Table 2:** Least square means for dam genotype (beef × beef, (B×B) and beef × dairy, (B×D)) and parity (primiparous and multiparous) on cow-calf maternal behaviours, colostrum IgG concentration, calf serum IgG concentration, ZST units and total protein (TP) concentration for experiment 1 (suckled calves) (*n* = 126)

	Genotype (G)		Parity (*P*)			*P* value^a^		
	B×B	B×D	Primiparous	Multiparous	Pooled SEM	G	P	
Time to first-licking the calf (sec)	70	84	98	56	14.1	NS	0.05	
Total duration of first-licking (sec)	77	75	42	110	16.2	NS	0.01	
Total number of attempts to stand by the calf	9	10	12	7	0.6	NS	0.001	
Total duration of the attempts to stand (sec)	84	93	76	100	15.4	NS	NS	
Time to calf standing on all fours (min)^a^	56	64	66	53	5.5	NS	NS	
Duration of the first standing on all fours (sec)	94	62	52	104	15.1	NS	0.05	
Total number of attempts to suckle before suckling occurred	10	11	13	8	0.8	NS	0.001	
Total duration of the attempts to suckle before suckling occurred (min)	4.6	5.7	4.6	5.7	0.61	NS	NS	
Time to first-suckle (min)	103	119	108	114	20.8	NS	NS	
Duration of the first-suckling bout (sec)	55	72	63	64	8.7	NS	NS	
Colostrum IgG (mg/ml)	132	119	117	134	5.8	NS	0.05	
Calf serum								
IgG (mg/ml)	47	47	44	50	2.8	NS	0.05	
ZST (units)	20	22	20	23	1.3	NS	0.05	
TP (g/dl)	6.0	6.0	5.8	6.2	0.15	NS	0.05	

^a^There was a genotype × parity interaction (*P* < 0.01) for time to standing (mean (SEM)) on all four legs (min). The respective values for B×B calves from multiparous were 52, (9.1) min and from primiparous cows were 60 (5.0) min, and for B×D calves from multiparous were 81 (10.8) min and from primiparous cows were 47 (4.8) min. There were no other genotype × parity interactions (*P* > 0.05) for maternal behaviours or for colostrum IgG concentration or calf passive immunity measures

### Passive immunity of suckled and artificially-fed calves

Overall mean (incorporating genotype and parity effects in the statistical model) colostrum IgG concentrations (SEM) (124 (4.1) vs. 120 (8.0) mg/ml)), and calf serum IgG concentrations (47 (1.8) and 44 (3.6) mg/ml), ZST units (21 (0.8) vs. 18 (1.8) units)) and TP concentrations (5.9 (0.1) vs. 5.5 (0.2) g/dl)) did not differ (*P* > 0.05) between suckled and artificially-fed calves, respectively (data not tabulated).

### Correlations between serum passive immunity tests and cow-calf maternal behaviour parameters (suckled)

There were positive spearman correlation coefficients for calf serum IgG with serum ZST (*r* = 0.65, *P* < 0.001) and serum TP (*r* = 0.57, *P* < 0.001), and for serum ZST with serum TP (*r* = 0.47, *P* < 0.001). There were no significant spearman correlation coefficients between cow-calf maternal behaviour and calf passive immunity measures (Table S1 supplementary file).

### Experiment 2

Cow live weight, BCS, docility score and calving difficulty score, and calf birth weight and calf vigour score of animals enrolled in Experiment 2 are described in Table 3.

**Table 3:** Means (SD) for beef × beef (B×B) and beef × dairy (B×D) cow weight, body condition score (BCS), docility score and calving difficulty score, and calf birth weight and calf vigour for the artificially-fed calves collected post-partum in experiment 2

	Beef × Beef cows	Beef × Dairy cows
Cow weight (kg)	542 (61.1)	547 (90.7)
Cow BCS (0–5)^1^	2.6 (0.18)	2.6 (0.18)
Cow docility score (1–5)^2^	1.3 (0.65)	1.1 (0.71)
Calving difficulty score (1–5)^3^	1.6 (0.93)	2.1 (1.19)
Calf birthweight (kg)	41.9 (6.02)	42.8 (7.21)
Calf vigour (1–5)^4^	4.2 (1.26)	4.5 (0.84)

^1^Cow BCS scale; 0–5 (0 = emaciated and 5 = very fat)

^2^Cow docility scale; 1–5; (1 = excellent and 5 = very aggressive)

^3^Calving difficulty score scale 1–5; (1 = no assistance; 2 = minor handling (‘checking’); 3 = minor assistance; 4 = mechanical assistance (calving jack) and 5 = caesarean section, the highest score was assigned to deliveries requiring veterinary assistance)

^**4**^Calf vigour scale; (1 = poor, 2 = very lazy, 3 = lazy, 4 = vigorous, and 5 = excellent/very vigorous)

### Cow genotype and parity effect on maternal bond formation, colostrum IgG concentration and calf passive immunity (artificially-fed calves)

The effect of genotype and breed on maternal behaviours and IgG concentration are presented in (Table 4). Calves from B×D cows had a greater (*P* < 0.05) mean number of attempts to suckle before suckling occurred compared to those from B×B cows. No effect (*P* > 0.05) of parity was found on the number of attempts to suckle. No effect (*P* > 0.05) of genotype or parity was found on the other cow-calf behaviours, or on colostrum IgG concentrations and calf passive immunity measures.

**Table 4:** Least square means for effect of dam genotype (beef × beef (B×B) *v.* beef × dairy (B×D)) and cow parity (parity 1 *v.* parity 2) on cow-calf maternal behaviours, colostrum IgG concentration, calf serum IgG concentration, ZST units and total protein (TP) concentration for experiment 2 (artificially-fed calves) (*n* = 60)

	Genotype (G)		Parity (*P*)		Pooled SEM	*P* value^a^		
	B×B	B×D	1	2		G	P	
Time to first-licking the calf (sec)^a^	29	25	52	40	13.5	NS	NS	
Total duration of first-licking (sec)	115	107	71	151	30.1	NS	NS	
Total number of attempts to stand by the calf	9	9	9	9	1.1	NS	NS	
Total duration of the attempts to stand (sec)	112	105	91	126	22.1	NS	NS	
Time to calf standing on all fours (min)	77	84	85	76	14.2	NS	NS	
Duration of the first-standing on all fours (sec)	287	163	165	284	78.7	NS	NS	
Total number of attempts to suckle before suckling occurred	4	6	5	5	0.7	0.05	NS	
Total duration of the attempts to suckle before suckling occurred (min)	2.7	2.9	2.5	3.1	0.49	NS	NS	
Time to first-suckle (min)	142	198	144	196	29.4	NS	NS	
Duration of the first-suckling bout (sec)	122	138	133	127	28.3	NS	NS	
Colostrum IgG (mg/ml)	122	127	122	127	7.9	NS	NS	
Calf serum								
IgG (mg/ml)	45	51	46	51	3.2	NS	NS	
ZST (units)	19	21	19	21	1.6	NS	NS	
TP (g/dl)	6.0	6.1	5.9	6.2	0.27	NS	NS	

There were no genotype × parity interactions (*P* > 0.05) for maternal behaviours or colostrum IgG concentration or calf passive immunity measures

### Correlations between calf passive immunity variables and cow-calf maternal behaviour parameters (artificially-fed)

There were positive spearman correlation coefficients for serum IgG with ZST (*r* = 0.69, *P* < 0.001), and TP (*r* = 0.59, *P* < 0.001), and for serum ZST with serum TP (*r* = 0.70, *P* < 0.001). There were no significant (*P* > 0.05) spearman correlation coefficients between serum passive immune tests and cow-calf maternal behaviour (Table S2 supplementary file).

## Discussion

The current study examined the effect of suckler cow genotype and parity on the development of the cow-calf bond, colostrum IgG concentrations and measures of calf passive immunity, and the relationship between cow-calf behaviours post-partum and calf passive immune status. In the present experiments, cows were nutritionally-managed to calve down at a moderate BCS [34], in order to reduce the incidence of calving difficulty. Beef calves born from dystocia births usually take longer to attempt to stand, stand successfully and to suckle compared to non-dystocic calves [26, 35], and are more likely to have lower or inadequate passive immunity [11, 18, 20, 36].

In Experiment 1, calves with a ‘strong’ suckle reflex were left to suckle naturally, whereas in the interests of health and welfare, calves with a ‘weak’ suckle reflex (mainly associated with a more difficult calving) were considered to be in need of assistance with colostrum feeding [19, 26, 36], and so were fed using an oesophageal-tube. Similarly, for possible health and welfare reasons, a cut-off of approximately 4-h was implemented regarding colostrum consumption for the calves with a ‘strong’ suckle reflex in Experiment 1, albeit only three calves exceeded this limit. In Experiment 2, the colostrum management regime entailed artificial-feeding with a stomach tube. Consequently, due to the ‘prophylactic nature’ of the colostrum intervention regimes employed, overall, this means that the influence of dystocia on cow-calf behaviour and subsequent passive immunity, as well as the impact of an excessive delay in colostrum consumption on passive immunity, is somewhat curtailed under the experimental conditions of this study.

### Maternal behaviour

There is comparatively little published literature focusing on the behaviour of suckler cows and their newborn calf immediately post-partum. Furthermore, direct comparison with literature is difficult due to differences in methodologies and metrics. In the current experiments, all multiparous cows licked their calves within the first minute post-partum, which broadly concurs with Cuttance et al. [37] who reported that 75% of spring-calving dairy cows calving outdoors in a calving paddock licked their calves within two minutes post-partum, and by six minutes post-partum this had increased to 95%. Vandenheede et al. [38] reported that the overall median time to first-licking of the newborn calf by Belgian Blue dams calving indoors was 3.3 min, and that the median latency to licking was numerically shorter in multiparous (2.3 min) compared to primiparous (11.7 min) animals. The earlier commencement of calf licking by multiparous (median 0.45 min) compared to primiparous (median 1.2 min) cows in Experiment 1, concurs with this. The relatively longer median latency reported by Vandenheede et al. [38] compared to the present study may be partly due to the fact that delivery was by caesarean section for the Belgian Blue cows. In dairy cows, Edwards and Broom [39] reported that licking bout-length during the first hour post-partum was 0.58 min, and did not differ between parity. This contrasts with multiparous cows in Experiment 1 having a longer duration (median, 1.3 min) of first-licking compared to primiparous cows (median, 0.62 min) cows. Considering that dairy calves are generally separated from their dam soon after birth, it is not overly-surprising that maternal responsiveness is more intense with suckler cows who have extended contact with their calves, and that the licking behaviour of an ‘experienced’ multiparous suckler cow is more persistent than a ‘naïve’ first-calver. The absence of a statistical effect of parity on cow licking behaviours in Experiment 2 compared to Experiment 1 likely reflects the colostrum-feeding regime employed in the former experiment.

In terms of calf behaviour, it is important to note that in Experiment 1, only the calves that ‘naturally’ suckled their dams within the first approximately 4-h post-partum were included in the behaviour observations. Consequently, by design, the ‘weak’ calves (i.e. poor suckle reflex) were excluded, which means that the calf vigour-related and ultimately colostrum suckling results obtained, are likely to be somewhat superior [26]. In Experiment 1, calves from primiparous cows made more attempts to stand before successfully standing compared to calves from multiparous cows, which is in contrast to the findings of Houwing et al. [40], who reported that calves from primiparous dairy cows calving indoors made fewer (12) attempts to stand before successfully standing compared to calves from multiparous cows [20]. This inconsistency may reflect dissimilarities in calving difficulty score between the parities across study, but also the differences in calf sire genotype between primiparous and multiparous cows in the current study. Mean times to first-standing in experiments 1 and 2 were intermediate to the range in mean values (30 min to 2 h) reported by Le Neindre and Vallet [41] in their review. In beef calves that were weighed, artificially-fed a colostrum product post-partum and subsequently placed in an individual pen with their dam, Gamsjäger et al. [42] reported that median latency to standing was 100 min (range; 15 to 614 min), which compares with the median time to standing of 62 min (range; 7 to 199 min) for B×B and 51 min (range; 7 to 241 min) for B×D calves, in Experiment 2.

Mean times from birth to first-suckling for experiments 1 and 2 are intermediate to the range in mean values (60 to 260 min) reported for beef calves in the review by Le Neindre and Vallet [41]. Langholz et al. [43] reported a large variation in time to first-suckling for individual calves ranging from 28 to 650 min, with a mean 269 min. Similarly, in Experiment 1 the range was 17 to 258 min, though by design the upper limit in our study was restricted to approximately 4 h and additionally the ‘weak’ calves (*n* = 23, 15%) were excluded as they were artificially-fed colostrum after birth. Unlike the present study where no effect of cow genotype was found on time to first-suckling, Langholz et al. [43] reported that first-suckling for calves from Simmental dams (297 min) accommodated in calving pens was later than those from Simmental × German-Friesian (246 min), with Charolais × German-Friesian (269 min) being intermediate. This disparity may reflect breed differences in maternal behaviour and other factors such as incidence of calving difficulty, across the studies. Also, unlike the current study, Langholz et al. [43] found that time to first-suckling was much later for calves from primiparous compared to multiparous (334 *v.* 202 min) cows which, in that study, was attributed to high-attached udders in primiparous dams impeding suckling. Vandenheede et al. [38] reported that the overall median time to first-suckling of Belgian Blue dams was 366 min, and that this was numerically shorter in multiparous (216 min) compared to primiparous (378 min) animals. Again, unlike the current experiments, delivery was by caesarean section in that study.

Although not directly comparable, it is noteworthy that time to first-suckling was much later in Experiment 2 compared to Experiment 1. This partially reflects the impact of the different methods of colostrum ingestion whereby the motivation to suckle may be curtailed in calves that are fed colostrum post-partum. In beef calves artificially-fed colostrum-product post-partum, Gamsjäger et al. [42] reported that the median time to first suckle was 162 min (range; 39 to 1440 min), which is longer than the median time to suckle of 94 min (range; 20 to 583 min) for calves in Experiment 2.

Langholz et al. [43] reported a mean duration of suckling until satiety of 19.0 to 22.8 min for calves from different suckler cow breeds, which is similar to the average total duration of suckling bouts including the rest times between bouts (not reported) in Experiment 1 for B×B calves (22 min; range, 1 to 133 min) and B×D calves (17 min; range, 2 to 64 min).

### Passive Immunity

Adequate passive transfer is dependent on several colostrum management factors, including colostrum Ig concentration, volume ingested, time of feeding and the calf’s ability to absorb Ig during the first 24-h of life [9]. The treatment mean colostrum IgG concentrations in the current experiments (119–132 mg/ml) are intermediate to the range in IgG(_1_) concentrations for suckler beef cows determined using RID in previous studies at this Research Centre (76–193 mg/ml: [1, 15, 21, 32], and in the recent international literature (84–146 mg/ml: [11, 23]).

The similar colostrum IgG concentrations obtained for B×B and B×D cows agrees with the findings of Murphy et al. [21] and Earley et al. [44]. The 15% greater (*P* = 0.05) colostrum IgG concentration in multiparous compared to primiparous cows in Experiment 1 is of the same magnitude reported by McGee et al. [15], although in the latter experiment the difference was not statistically significant. Similarly, in Experiment 2, there was only a 4% numerical difference in favour of the ‘older’ dams, although these cows were only second-parity rather than multiparous animals. In their review of the literature, McGee and Earley [9] reported that unlike dairy cows differences in colostrum immunoglobulin concentrations between primiparous and multiparous beef-suckler cows are generally relatively small and often do not differ statistically. More recently, Altvater-Hughes et al. [23] also reported that colostrum IgG concentrations increased with parity in dairy cows but not in beef cows.

The range in mean IgG concentrations (44–51 mg/ml) in the current experiments are intermediate (34.4–62.4 mg/ml [1, 15, 32]) or higher (18.1–27.1 mg/ml [21]) than IgG_1_ concentrations measured using RID in previous studies at this Research Centre. They are also higher than recent international studies using RID for determining beef calf serum IgG_1_ concentrations on commercial (35.2, mg/ml [19]; 43.3 mg/ml [36]; 39.9 mg/ml [20]) or experimental research farms (19.9 mg/ml [11]). Using serum IgG cut-offs of < 10 mg/ml for FTPI classification, and < 24 mg/ml for having inadequate passive immunity (e.g. Gamsjäger et al. [20], in Experiment 1 the percentage of ‘natural-suckling’ calves falling into these two classifications was 0.8 and 7.1, respectively. Corresponding values for the artificially-fed calves was 0% and 15.4%. In Experiment 2, the percentage of calves (all artificially-fed) falling into these two classifications was 1.7 and 6.7, respectively. These percentages are much lower than reported for commercial beef farms in Ireland [10], Great Britain [18] and Canada [19, 20], and research farms in France [11]. This disparity can be partly attributed to the prophylactic nature of the colostrum feeding regime imposed in the current study (i.e. early ingestion of an adequate volume of first-milking colostrum from the dam), as discussed earlier, but also the vagaries associated with the accuracy and precision of methodologies used to determine Ig concentrations [9]. Nevertheless, collectively the findings in the current study indicate that passive immunity in suckler calves is largely successful when early colostrum-feeding intervention, based on scoring calf vigour, occurs.

The similarity in calf passive immunity measures between suckled and artificially-fed calves in Experiment 1, concurs with McGee et al. [15] who found no difference in calf serum IgG concentrations between calves assisted to suckle or fed a colostrum volume equivalent to 5% of their birth weight using an oesophageal tube, within 1-hour of birth.

In the present experiments, no effect of cow genotype was found on serum IgG concentrations, ZST units or TP concentrations, whereas in previous studies at Teagasc Grange, Murphy et al. [21] and McGee et al. [1] found that B×D calves had greater serum IgG concentrations and ZST units compared to B×B calves under natural suckling conditions. Similarly, in New Zealand, Hickson et al. [25] reported that Angus × Holstein-Friesian calves had greater serum IgG and TP concentrations compared to pure-bred Angus calves. The discrepancy across studies and convergence of the genotypes may be partly attributed to the fact that, unlike previous Irish experiments, the BxB cows in the present study were bred for improved maternal traits, particularly milk yield [3]. Calves from cow genotypes with higher milk yield also have higher passive immune status [21], likely due to a greater colostrum yield and thus Ig mass production [1]. Although dairy calves generally have an inferior passive immune status than suckler calves under controlled research conditions [9, 32], Todd et al. [10] reported greater serum IgG concentrations and ZST units, and similar TP concentrations in dairy calves than suckler beef calves on Irish commercial farms. The latter differences may be a reflection of calf management practice whereby dairy calves are typically removed soon after birth and are artificially-fed colostrum, whereas suckler calves remain with the cow to suckle naturally [9]; however, it may also suggest that, nationally, genotype differences are converging. The serum IgG concentrations, ZST units and TP concentrations in calves from multiparous cows compared to primiparous cows in Experiment 1, concurs with previous research [15, 44]. This is likely attributed to a greater colostrum yield and Ig mass, produced by multiparous compared to primiparous cows [15]. The absence of an effect of parity in Experiment 2 is very likely because of the colostrum-feeding regime employed, as discussed earlier.

In Experiments 1 and 2, there were no correlations of behavioural parameters with calf passive immunity measures for the naturally suckled calves or for artificially-fed calves, which is in agreement with Ruiz et al. [45]. Although it is well recognised that increasing the duration between birth and first suckling negatively impacts calf passive immunity McGee and Earley [9], the absence of a relationship between time to first suckle and calf serum IgG levels is likely due to the colostrum management regimen, whereby high-risk calves (i.e. weak suckle reflex) were artificially-fed and a time limit to suckling of 4 h was imposed. These interventions would be considered to be good husbandry practice to ensure good calf health and welfare.

In both experiments, serum IgG concentrations were strongly correlated with ZST units and TP concentrations. The correlations for serum IgG with ZST units (*r* = 0.65 and *r* = 0.69, in experiments 1 and 2, respectively) are in agreement with the findings (*r* = 0.78) of Dunn et al. [32]. Likewise, the correlation (*r* = 0.53) for serum ZST units with TP concentrations found by Todd et al. [10] is intermediate to the values found in experiment 1 (*r* = 0.47) and lower than in experiment 2 (*r* = 0.70). The correlations for serum IgG with TP concentrations in experiments 1 (*r* = 0.57) and 2 (*r* = 0.59) are lower than found by Todd et al. [10] comparing TP with ELISA IgG (*r* = 0.64); by Keuder et al. [46] (*r* = 0.75), Calloway et al. [47], by Pisello et al. [48], Gamsjäger et al. [49] comparing TP using three (*r* = 0.74–0.77), four (*r* = 0.75–0.84), and three (0.82 to 0.91) different refractometers, respectively, by Akköse et al. [50] (0.877) comparing TP with sRID (*r* = 0.877), and by Vandeputte et al. [51] comparing TP using four different refractometers (0.77–0.82) instead of HPLC IgG.

This disparity across studies may be attributed to differences in the methodologies used, but also due to the fact that the mean sRID IgG concentrations (44–51 mg/ml) in the present study are substantially higher than reported by Todd (12 mg/ml) [10], Calloway et al. (10 mg/ml) [47], Kreuder et al. (34.4 mg/ml) [46], Pisello et al. (16.9 mg/ml) [48] and a linear relationship across very diverse IgG concentrations for TP may not apply. Although RID is the ‘gold standard’ for measuring IgG concentrations in calf serum [1, 10, 18], other laboratory- based tests such as Zinc Sulphate Turbidity (ZST) have been used previously on suckler calves [1, 10, 52]. More recently, cheaper, more rapid pen-side tests to measure passive immunity such as total protein (TP) (by refractometry) are reported in the literature for dairy calves [10, 47, 53, 54] and suckler calves [10, 47, 48, 51].

## Conclusion

Overall, there was little effect of cow genotype on cow-calf behaviours but under ‘natural suckling’ conditions primiparous cows expressed maternal inexperience and their calves were less vigorous compared to multiparous cows. Colostrum IgG concentrations and calf passive immunity measures were unaffected by cow genotype, but under ‘natural suckling’ conditions calves from primiparous cows had lower passive immunity. The findings from this study indicate that greater vigilance is required for primiparous suckler cows and their calves compared to multiparous cows in relation to calf passive immune status. Furthermore, the current results indicate that passive immunity in suckler calves is largely successful when colostrum-feeding intervention, based on scoring calf vigour (‘suckle reflex’), is implemented.

### Electronic supplementary material

Below is the link to the electronic supplementary material.

Supplementary Material 1

Supplementary Material 2

## Data Availability

All data supporting the research findings are included within the manuscript. The data are available from the corresponding author upon request.

## Abbreviations

B×B: beef × beef
BCS: Body condition score
B×D: beef × dairy
CCTV: Close circuit television
d: Day
DM: Dry matter
FTPI: Failed transfer of passive immunity
G: Genotype
h: hour
HPLC: High performance liquid chromatography
IgG: Immunoglobulin G
Min: Minute
Ml: Millilitre
P: Parity
sRID: single radial immunodiffusion
TP: Total protein
V: Versus
ZST: Zinc sulphate turbidity

## References

[1] McGee M, Drennan MJ, Caffrey PJ (2005). Effect of suckler cow genotype on cow serum immunoglobulin (ig) levels, colostrum yield, composition and ig concentration and subsequent immune status of their progeny. Ir J Agric Food Res.

[2] McCabe S, Prendiville R, Evans R, O’Connell NE, McHugh N (2019). Effect of cow replacement strategy on cow and calf performance in the beef herd. Animal.

[3] McCabe S, McHugh N, O’Connell NE, Prendiville R (2021). Performance of lactating suckler cows of diverse genetic merit and genotype under a seasonal pasture-based system. Ir J Agric Food Res.

[4] von Keyserlingk MAG, Weary DM (2007). Maternal behavior in cattle. Horm Behav.

[5] Whalin L, Weary DM, von Keyserlingk MAG (2021). Understanding behavioural development of calves in natural settings to inform Calf Management. Anim (Basel).

[6] Nevard RP, Pant SD, Broster JC, Norman ST, Stephen CP (2022). Maternal behavior in beef cattle: the physiology, assessment and future directions-A review. Vet Sci.

[7] Jensen MB (2011). The early behaviour of cow and calf in an individual calving pen. Appl Anim Behav Sci.

[8] Johnsen JF, de Passille AM, Mejdell CM, Bøe KE, Grøndahl AM, Beaver A, Rushen J, Weary DM (2015). The effect of nursing on the cow-calf bond. Appl Anim Behav Sci.

[9] McGee M, Earley B (2019). Review: passive immunity in beef-suckler calves. Animal.

[10] Todd CG, McGee M, Tiernan K, Crosson P, O’Riordan E, McClure J, Lorenz I, Earley B (2018). An observational study on passive immunity in Irish suckler beef and dairy calves: tests for failure of passive transfer of immunity and associations with health and performance. Prev Vet Med.

[11] Martin P, Vinet A, Denis C, Grohs C, Chanteloup L, Dozias D, Maupetit D, Sapa J, Renand G, Blanc F (2021). Determination of immunoglobulin concentrations and genetic parameters for colostrum and calf serum in Charolais animals. J Dairy Sci.

[12] Gamsjäger L, Haines DM, Lévy M, Windeyer MC (2023). Total and pathogen-specific serum immunoglobulin G concentrations in neonatal beef calves, part 2: associations with health and growth. Prev Vet Med.

[13] Raboisson D, Trillat P, Cahuzac C (2016). Failure of passive immune transfer in calves: a meta-analysis on the consequences and assessment of the economic impact. PLoS ONE.

[14] Weaver DM, Tyler JW, VanMetre DC, Hostetler DE, Barrington GM (2000). Passive transfer of colostral immunoglobulins in calves. J Vet Intern Med.

[15] McGee M, Drennan MJ, Caffrey PJ (2006). Effect of age and nutrient restriction pre partum on beef suckler cow serum immunoglobulin concentrations, colostrum yield, composition and immunoglobulin concentration and immune status of their progeny. Ir J Agric Food Res.

[16] Pearson JM, Pajor EA, Caulkett NA, Levy M, Campbell JR, Windeyer MC (2019). Benchmarking calving management practices on western Canada cow–calf operations. Transl Anim Sci.

[17] Desjardins-Morrissette M, van Niekerk JK, Haines D, Sugino T, Oba M, Steele MA. 2018. The effect of tube versus bottle feeding colostrum on immunoglobulin G absorption, abomasal emptying, and plasma hormone concentrations in newborn calves. J Dairy Sci. 2021; 101: 4168-79. 10.3168/jds.2017-13904 29454696

[18] Bragg R, Macrae A, Lycett S, Burrough E, Russell G, Corbishley A (2020). Prevalence and risk factors associated with failure of transfer of passive immunity in spring born beef suckler calves in Great Britain. Prev Vet Med.

[19] Pearson JM, Pajor EA, Campbell JR, Levy M, Caulkett NA, Windeyer MC (2019). A randomised controlled trial investigating the effects of administering a non-steroidal anti-inflammatory drug to beef calves assisted at birth and risk factors associated with passive immunity, health, and growth. Vet Rec Open.

[20] Gamsjäger L, Haines DM, Lévy M, Windeyer MC (2023). Total and pathogen-specific serum immunoglobulin G concentrations in neonatal beef calves, part 1: risk factors. Prev Vet Med.

[21] Murphy BM, Drennan MJ, O’Mara FP, Earley B (2005). Cow serum and colostrum immunoglobulin (IgG1) concentration of five suckler cow breed types and subsequent immune status of their calves. Ir J Agric Food Res.

[22] Vandeputte S, Detilleux J, Rollin F. Investigation of colostrum quality in beef cattle by radial immunodiffusion and brix refractometry. Vet Rec. 2014;175(14):353. 10.1136/vr.101590. Epub 2014 Aug 5. PMID: 25096590. 10.1136/vr.101590 25096590

[23] Altvater-Hughes TE, Hodgins DC, Wagter-Lesperance L, Beard SC, Cartwright SL, Mallard BA (2022). Concentration and heritability of immunoglobulin G and natural antibody immunoglobulin M in dairy and beef colostrum along with serum total protein in their calves. J Anim Sci.

[24] Earley B, McGee M, Fallon RJ, Drennan MJ, Murray M, Farrell JA (2000). Serum immunoglobulin concentrations in suckled calves and dairy-herd calves. Ir J Agricultural Food Res.

[25] Hickson R, Back P, Martin N, Kenyon P, Morris S (2016). The influence of age and breed of cow on colostrum indicators of suckled beef calves. Proc New Zeal Soc Anim Prod.

[26] Homerosky ER, Timsit E, Pajor EA, Kastelic JP, Windeyer MC (2017). Predictors and impacts of colostrum consumption by 4 h after birth in newborn beef calves. Vet J.

[27] Berry DP, Amer PR, Evans RD, Byrne T, Cromie AR, Hely F (2019). A breeding index to rank beef bulls for use on dairy females to maximize profit. J Dairy Sci.

[28] ICBF. 2017. Irish Cattle Breeding Federation Annual Report 2017. Recording birth events. Highfield House, Shinagh, Bandon, Co. Cork, Ireland (accessed 28 March 2024, https://www.icbf.com/wp-content/uploads/2013/06/Birth-events.pdf).

[29] Lowman BG, Scott NA, Somerville SH. Condition scoring of cattle. East of Scotland College of Agriculture. Animal Production, Advisory and Development Department Edinburgh: (1976) Edinburgh School of Agriculture Bulletin, ISBN 0902164236, 9780902164239 No. 6.

[30] Stěhulová I, Špinka M, Šárová R, Máchová L, Kněz R, Firla P (2013). Maternal behaviour in beef cows is individually consistent and sensitive to cow body condition, calf sex and weight. Appl Anim Behav Sci.

[31] McEwan AD, Fisher EW, Selman IE, Penhale WJ (1970). A turbidity test for the estimation of immune globulin levels in neonatal calf serum. Clin Chim Acta.

[32] Dunn A, Duffy C, Gordon A, Morrison S, Argűello A, Welsh M, Earley B (2018). Comparison of single radial immunodiffusion and ELISA for the quantification of immunoglobulin G in bovine colostrum, milk and calf sera. J Appl Anim Res.

[33] SAS Institute Inc, Cary N, USA. SAS^®^9.4 Language Reference: Concepts, Sixth Edition. Volume SAS. Analytics, Artificial Intelligence and Data Management; 2016.

[34] Drennan MJ, McGee M (2004). Effect of suckler cow genotype and nutrition level during the winter on voluntary intake and performance and on the growth and slaughter characteristics of their progeny. Ir J Agric Food Res.

[35] Hickson RE, Lopez-Villalobos N, Kenyon PR, Morris ST (2008). Duration of parturition and frequency of abdominal contractions in primiparous, 2-year-old Angus heifers and the relevance of body dimensions of calves to dystocia. Aust J Exp Agric.

[36] Pearson JM, Homerosky ER, Caulkett NA, Campbell JR, Levy M, Pajor EA, Windeyer MC (2019). Quantifying subclinical trauma associated with calving difficulty, vigour, and passive immunity in newborn beef calves. Vet Rec Open.

[37] Cuttance EL, Mason WA, McDermott J, Laven RA (2022). Suckling behavior of calves in seasonally calving pasture-based dairy systems, and possible environmental and management factors affecting suckling behaviors. J Dairy Sci.

[38] Vandenheede M, Nicks B, Désiron A, Canart B (2001). Mother–young relationships in Belgian blue cattle after a caesarean section: characterisation and effects of parity. Appl Anim Behav Sci.

[39] Edwards SA, Broom DM (1982). Behavioural interactions of dairy cows with their newborn calves and the effects of parity. Anim Behav.

[40] Houwing H, Hurnik JF, Lewis NJ (1990). Behavior of periparturient dairy cows and their calves. Can J Anim Sci.

[41] Le Neindre P, Vallet A, Jarrige R, Beranger C (1992). The suckled calf. Beef cattle production, World Animal Science.

[42] Gamsjäger L, Haines DM, Pajor EA, Lévy M, Windeyer MC (2021). Impact of volume, immunoglobulin G concentration, and feeding method of colostrum product on neonatal nursing behaviour and transfer of passive immunity in beef calves. Animal.

[43] Langholz HJ, Schmidt FW, Derenbach J, Kim JW (1987). Suckling behaviour, immunglobulin status and weaning performance in suckler cows. World Rev Anim Prod.

[44] Earley B, Tiernan K, Duffy C, Dunn A, Waters S, Morrison S, McGee M (2018). Effect of suckler cow vaccination against glycoprotein E (gE)-negative bovine herpesvirus type 1 (BoHV-1) on passive immunity and physiological response to subsequent bovine respiratory disease vaccination of their progeny. Res Vet Sci.

[45] Ruiz L. 2019. Behavioral biomarkers for calf health. Master of Science, Kansas State University. Accessed 28th March 2024, https://krex.k-state.edu/bitstream/handle/2097/39550/LukeRuiz2019.pdf?sequence=3.

[46] Kreuder AJ, Breuer RM, Wiley C, Dohlman T, Smith JS, McKeen L (2023). Comparison of turbidometric immunoassay, refractometry, and gamma-glutamyl transferase to radial immunodiffusion for assessment of transfer of passive immunity in high-risk beef calves. J Vet Intern Med.

[47] Calloway CD, Tyler JW, Tessman RK, Hostetler D, Holle J (2002). Comparison of refractometers and test endpoints in the measurement of serum protein concentration to assess passive transfer status in calves. J Amer Vet Med Assoc.

[48] Pisello L, Boccardo A, Forte C, Pravettoni D, D’Avino N, Passamonti F, Rueca F. 2021, Evaluation of digital and optical refractometers for assessing failure of transfer of passive immunity in Chianina beef–suckler calves reared in Umbria. Italian J Anim Sci. 2021; 20:1, 315–323.

[49] Gamsjager L, Elsohaby I, Pearson JM, Levy M, Pajor EA, Windeyer MC (2021). Evaluation of 3 refractometers to determine transfer of passive immunity in neonatal beef calves. J Vet Intern Med.

[50] Akköse M, Özbeyaz C, Buczinski S. 2022. Evaluation of 2 refractometers to estimate different passive immunity status in Simmental dairy calves, preventive Veterinary Medicine, 209, 105778, ISSN 0167–5877, 10.1016/j.prevetmed.2022.105778. 10.1016/j.prevetmed.2022.105778 36279662

[51] Vandeputte S, Detilleux J, Rollin F (2011). Comparison of four refractometers for the investigation of the passive transfer in beef calves. J Vet Intern Med.

[52] O’Shaughnessy J, Earley B, Barrett D, Doherty ML, Crosson P, de Waal T, Mee JF (2015). Disease screening profiles and colostrum management practices on 16 Irish suckler beef farms. Ir Vet J.

[53] Lombard J, Urie N, Garry F, Godden S, Quigley J, Earleywine T, McGuirk S, Moore D, Branan M, Chamorro M, Smith G, Shivley C, Catherman D, Haines D, Heinrichs AJ, James R, Maas J, Sterner K (2020). Consensus recommendations on calf- and herd-level passive immunity in dairy calves in the United States. J Dairy Sci.

[54] Cuttance EL, Regnerus C, Laven RA (2019). A review of diagnostic tests for diagnosing failure of transfer of passive immunity in dairy calves in New Zealand. N Z Vet J.

