# Supplementary material for: Effect of suckler cow breed type and parity on the development of the cow-calf bond post-partum and calf passive immunity
Source: Ir Vet J. 2024 Jul 5;77:13. doi: 10.1186/s13620-024-00276-x (PMC11225149; doi:10.1186/s13620-024-00276-x)
Supplement: Supplementary file 1 — Supplementary Material 1 [file 13620_2024_276_MOESM1_ESM.docx]

**Table S1** Spearman correlation coefficients between cow-calf maternal behaviours and calf passive immunity parameters (Experiment 1 suckled)

(Supplementary file S1)

|  | Calf passive immunity measures | | |
| --- | --- | --- | --- |
| Cow-calf behaviours | IgG | ZST | TP |
| Time to first-licking the calf (sec) | 0.05 | 0.02 | 0.02 |
| Total duration of first-licking (sec) | 0.19 | 0.21 | 0.16 |
| Total number of attempts to stand | -0.04 | -0.04 | 0.01 |
| Total duration of the attempts to stand (sec)^a^ | 0.16 | 0.16 | 0.13 |
| Time to standing on all fours (min) | 0.06 | -0.12 | -0.04 |
| Duration of the first standing on all fours (sec) | 0.12 | -0.05 | -0.03 |
| Total number of attempts to suckle before suckling occurred (sec) | 0.05 | 0.02 | 0.07 |
| Total duration of attempts to suckle before suckling occurred (min) | 0.13 | 0.08 | -0.003 |
| Time to first-suckle (min) | -0.12 | -0.20 | 0.022 |
| Duration of the first-suckling bout (sec) | 0.15 | 0.13 | 0.14 |
