# Supplementary material for: Effect of suckler cow breed type and parity on the development of the cow-calf bond post-partum and calf passive immunity
Source: Ir Vet J. 2024 Jul 5;77:13. doi: 10.1186/s13620-024-00276-x (PMC11225149; doi:10.1186/s13620-024-00276-x)
Supplement: Supplementary file 2 — Supplementary Material 2 [file 13620_2024_276_MOESM2_ESM.docx]

**Table S2** Spearman correlation coefficients between cow-calf maternal behaviours and calf passive immunity parameters (Experiment 2 artificially-fed).

**Supplementary file S2**

|  | Calf passive immunity measures | | |
| --- | --- | --- | --- |
| Cow-calf behaviours | IgG | ZST | TP |
| Time to first-licking the calf (sec) | 0.12 | -0.06 | -0.03 |
| Total duration of first-licking (sec) | 0.07 | -0.07 | -0.16 |
| Total number of attempts to stand | -0.22 | 0.007 | -0.06 |
| Total duration of the attempts to stand (sec) | 0.04 | 0.10 | -0.02 |
| Time to standing on all fours (min) | -0.05 | 0.01 | -0.10 |
| Duration of the first-standing on all fours (sec) | -0.56 | 0.18 | 0.09 |
| Total number of attempts to suckle before suckling occurred (sec) | -0.22 | -0.05 | -0.03 |
| Total duration of the attempts to suckle before suckling occurred (min) | -0.18 | -0.11 | -0.15 |
| Time to first-suckle (min) | -0.09 | -0.10 | -0.18 |
| Duration of the first-suckling bout (sec) | 0.24 | 0.04 | -0.002 |
